# Expanding the Mutation Spectrum for Inherited Retinal Diseases

**DOI:** 10.3390/genes16010032

**Published:** 2024-12-28

**Authors:** Jacob Lynn, Samuel J. Huang, Grace K. Trigler, Ronald Kingsley, Razek G. Coussa, Lea D. Bennett

**Affiliations:** 1 Department of Pathology, University of Oklahoma Health Sciences Center, Oklahoma City, OK 73114, USA; jacob-lynn@ouhsc.edu; 2 Department of Ophthalmology, University of Oklahoma Health Sciences Center, Oklahoma City, OK 73114, USA; samuel-huang@ouhsc.edu (S.J.H.); grace-trigler@ouhsc.edu (G.K.T.); 3 Dean McGee Eye Institute, Oklahoma City, OK 73140, USA; ronald-kingsley@dmei.org (R.K.); rgcoussa@rvcow.com (R.G.C.)

**Keywords:** inherited retinal disease, genetic testing, genotype, phenotype

## Abstract

Background/Objectives: Inherited retinal diseases (IRDs) represent a diverse group of genetic disorders characterized by degeneration of the retina, leading to visual impairment and blindness. IRDs are heterogeneous, sharing common clinical features that can be difficult to diagnose without knowing the genetic basis of the disease. To improve diagnostic accuracy and advance understanding of disease mechanisms, genetic testing was performed for 103 unrelated patients with an IRD at a single clinical site between 30 August 2022 and 5 February 2024. Methods: Informed consent was obtained before buccal samples were collected for panel-based sequencing at BluePrint Genetics (BpG), sponsored by the Foundation Fighting Blindness MyRetina Tracker program. A retina specialist performed standard visit assessments, including visual acuity (Snellen chart), slit lamp examination, fundus photography (Optos^®^, Dunfermline, UK), and spectral-domain optical coherence tomography (SD-OCT; Zeiss). Results: From 103 patients, genetic findings were reported for 70 individuals. Among these included 20 novel variants. Conclusions: These results clarify and confirm clinical diagnoses, aid in counseling patients on prognosis and family planning, and guide treatment options. This study not only holds promise for affected individuals but also expands the mutation spectrum to guide understanding of IRD.

## 1. Introduction

Inherited retinal diseases (IRDs) represent a diverse group of genetic disorders affecting the photoreceptor cells and/or the retinal pigment epithelium (RPE). They are characterized by progressive vision loss and retinal degeneration that occur in 1:3000 individuals worldwide [1,2,3,4]. These conditions show significant clinical and genetic heterogeneity, with symptoms, severity, and progression varying widely, even within the same family. IRDs are broadly categorized into three clinical types [4]. The most common is the photoreceptor disease type, in which retinitis pigmentosa (RP) predominates, but also includes IRDs such as cone and cone-rod dystrophy (CD/CRD) and Leber congenital amaurosis (LCA). These can be further subdivided based on the primary photoreceptor affected. Rod-dominant IRDs, such as retinitis pigmentosa (RP), typically begin with night blindness and peripheral degeneration, progressing to a constricted visual field and central vision loss [5]. In contrast, cone-dominant IRDs, like CRD and achromatopsia, are characterized by decreased visual acuity, impaired color vision, and light sensitivity. The second is the macular disease type, which primarily affects the RPE, leading to secondary photoreceptor loss in the macular region. IRDs within this category include Best disease, pattern dystrophy, and Stargardt disease (STGD1). The final category is referred to as third-branch disorders, including IRDs like choroideremia, optic neuropathy, and X-linked retinoschisis (XLRS). This categorization is not always straightforward, as many IRDs have overlapping symptoms. This is especially true as diseases progress and multiple cell types can become involved. Further, IRDs can be described by their inheritance pattern: autosomal dominant, autosomal recessive, or X-linked. Phenotypic overlap complicates accurate diagnosis and emphasizes the need for genetic testing to accompany clinical evaluation when diagnosing IRDs.

An accurate clinical diagnosis supported by patients’ genetics equips physicians to better treat and counsel patients on prognosis and family planning. Genetically, IRDs are associated with mutations in at least 326 genes (https://web.sph.uth.edu/RetNet/ accessed on 27 November 2024). Since the approval of Luxturna, a gene therapy that targets biallelic *RPE65* mutations [6], there has been a surge in preclinical and clinical gene therapy trials to treat IRD [7]. The objectives of this study were to enhance diagnostic accuracy, identify factors that affect negative genetic results, and deepen the understanding of disease mechanisms in IRDs. Toward this goal, we analyzed genetic data from 103 unrelated patients, identifying genetic causes in 70 cases, including 20 novel variants that supported diagnosis, informed patient counseling, and provided treatment guidance. The results highlight the diversity of the mutational spectrum of IRDs, identify factors influencing negative genetic results, and expand the clinical phenotype associated with the genes *ALMS1*, *GNAT1*, *RAX2*, and *RDH5*.

## 2. Materials and Methods

The unrelated patients (n = 103; 53 = male, 51.5%) were referred from an outside provider and seen at Dean McGee Eye Institute (DMEI) at the University of Oklahoma Health Sciences Center (OUHSC), the leading diagnostic center for IRDs in Oklahoma, by a retinal and IRD specialist (R.K. or R.G.C.). Based on clinical presentation, patient history, and additional testing, including multimodal imaging, visual fields, and electroretinography, the patients were diagnosed with an IRD by the specialists mentioned and were recruited for genetic testing. All procedures adhered to the Declaration of Helsinki and were approved by the Institutional Review Board (IRB). Patients were excluded if they had been diagnosed with autoimmune retinopathy, chorioretinal infection, or age-related macular degeneration. Also, patients taking retinotoxic medications (e.g., pentosan polysulfate or hydroxychloroquine) were excluded.

Patients received a standard ophthalmic exam as well as visual acuity and imaging. At the visit, we obtained a buccal swab and information including race (self-reported), age of onset (defined as self-reported age at the onset of the first symptom) (Table 1), and family history from each patient (to be used in conjunction with a genetic report to determine inheritance pattern). Visual acuity was measured according to Early Treatment of Diabetic Retinopathy Study (ETDRS) [8] guidelines using a traditional Snellen chart. Acuity was converted to a logarithm of minimal angle of resolution (logMAR). Fundus images were acquired with the Optos^®^ camera (Optos PLC, Dunfermline, UK). Horizontal line scans were obtained with spectral-domain optical coherence tomography (SD-OCT; Carl Zeiss Meditec USA, Inc.). Genetic testing was performed after obtaining informed, written consent. Buccal swab samples were collected using the DNAgenotek OCD-100 collection kit and submitted through the MyRetina Tracker (MRT) Program for analysis using gene panel-based sequencing with Blueprint Genetics (BpG) Laboratory (Marlborough, MA, USA), sponsored by Foundation Fighting Blindness (FFB). The 351 gene panel included the known non-coding IRD variants and the mitochondrial genome and is further detailed at the BpG website www.blueprintgenetics.com. Exam findings accompanied the DNA sample for variant interpretation by the geneticist at BpG. Sequence variants classified as pathogenic, likely pathogenic, and variants of uncertain significance (VUS) reported as primary findings on the patients’ genetic report (by certified geneticists at BpG) were included in this study. The pathogenicity of the variants was determined according to ACMG guidelines by BpG using their in-house algorithm, which is publicly available on the BpG website www.blueprintgenetics.com. Patients received genetic counseling through Informed DNA, sponsored by MRT-FFB.

## 3. Results

### 3.1. Cohort Characteristics and Molecular Diagnosis

Diagnostic groups were based on the Iowa classification of IRD [4]. The majority of patients (60%, n = 62) had photoreceptor disease, the most common being RP (n = 57). Four patients were diagnosed with CD/CRD, a cone-dominant photoreceptor disease type. The patients with macular diseases (35%, n = 36) included macular dystrophy (MD, n = 18), pattern dystrophy (PD, n = 2), Best disease (n = 5), and STGD1 (n = 11). Other inherited diseases in this cohort included third-branch disorders (5%, n = 5), such as familial exudative vitreoretinopathy (FEVR, n = 2) and X-linked retinoschisis (XLRS, n = 3).

**Figure 1:**
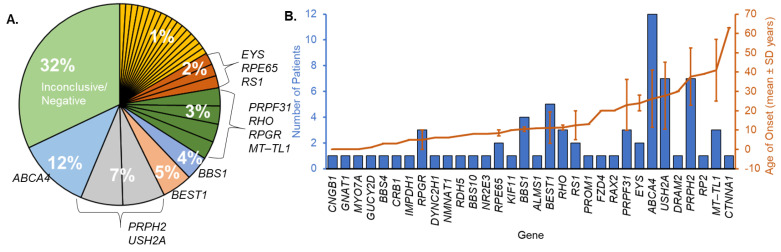
Gene frequency and age of onset. (**A**) Percentage of cases associated with identified genes. Genes associated with one patient (yellow slices) each were *BBS10*, *BBS4*, *CNGB1*, *CRB1*, *FZD4*, *GNAT1*, *IMPDH1*, *KIF11*, *MYO7A*, *NMNAT1*, *NR2E3*, *PROM1*, *RAX2*, *RDH5*, and *RP2.* (**B**) Mean ± SD age of onset is indicated by the orange trend line. The number of patients diagnosed per gene is indicated by the blue bars.

The primary findings from genetic testing revealed pathogenic variants in 29 genes for 70 out of 103 patients (68%; Table 1). The most commonly identified gene was ABCA4 (n = 12; Figure 1A). PRPH2 (n = 7) and USH2A (n = 7) were the next most prevalent genes containing pathogenic variants identified in this cohort. Five patients had causative variants in BEST1, and four patients with Bardet–Biedl syndrome (BBS) had variants in BBS1. Two other patients with BBS harbored variants in BBS4 or BBS10. Overall, 16 syndromic IRDs were documented for this cohort (Table 1).

Although all the patients in the cohort had an IRD diagnosis, 32% (n = 33) of patients received negative genetic testing results. To understand the factors influencing these negative results, we evaluated whether they were an isolated case within their family and age of onset. Twenty-eight of these patients reported no family history of a similar eye disease. Additionally, the age of onset for the 33 patients with negative findings was 34 ± 19 years, which was significantly older than the age of those for whom we identified causative mutation(s) (19 ± 16 years, *p* < 0.0005; Figure 1B). The percentage of patients in the negative genetic group with an age of onset greater than 40 years was 51.5%, but only 10% of patients with causative genetic mutations were 40 years or older.

### 3.2. Novel Genetics and Clinical Phenotypes

From 70 positive genetic results, 20 novel variants were identified in 19 patients. Novel variants were designated as being absent in the literature or not found in large population databases such as the Genome Aggregation Database (gnomAD) within the patients’ genetic report created by the geneticists at BpG. These included 14 missenses, two duplications, one insertion–deletion (indel), one copy number variant (CNV), one nonsense, and two frameshift mutations (Table 1). Variant modeling and ACMG criteria for pathogenicity are provided in Table 2. Four of these novel variants were homozygous recessive, nine were heterozygous recessive but paired with a second pathogenic or likely pathogenic mutation in the same gene, and seven were specific to dominant IRDs. The clinical phenotypes associated with these novel variants are provided in the Supplemental Table. The clinical characteristics linked to mutation in the genes *BEST1* [9,10,11,12,13,14,15,16,17,18,19,20,21], *BBS1* [22,23,24,25,26,27,28,29,30,31,32], *BBS4* [33,34], *BBS10* [35,36,37,38], *EYS* [4,39,40,41,42], *GUCY2D* [43,44,45,46,47], *PRPH2* [48,49,50,51,52,53,54], *RHO* [55,56,57,58], *RP2* [59,60,61], and *USH2A* [62,63,64,65,66,67,68,69,70,71,72,73,74] have been well described in the literature. Our patients displayed typical clinical features such as Bestrophinopathy (*BEST1*), pattern dystrophy (*PRPH2*), and BBS (*BBS1*, *BBS4*, *BBS10*). Those with RP showed varying degrees of bone spicule pigmentation, pallor of the optic disc, and vessel attenuation. However, the novel variants in the genes *ALMS1*, *GNAT1*, *RAX2*, and *RDH5* represent ultra-rare conditions with sparse clinical data available in the literature. These are described below.

#### 3.2.1. *ALMS1*

GP2-41 is an 11-year-old male with Alström syndrome, an autosomal recessive multisystem disorder that manifested in this patient with obesity, fatty liver, prediabetes, hyperinsulinemia, and hearing loss. Family history was limited due to the patient’s adoption, but the patient appears to be an isolated case of Alström syndrome. GP2-41 was referred for a retina evaluation that demonstrated the normal appearance of the fundus but disruption of the ellipsoid zone on SD-OCT in both eyes. A homozygous VUS c.103_108dup, p.(Ala35_Ala36dup) was identified by genetic testing in the gene *ALMS1*. This variant results in the duplication of two amino acids with preservation of the reading frame and has previously been reported to ClinVar (variation ID 403944). These amino acid positions are not well conserved across vertebrate species to *Danio rerio* (zebrafish). The duplication affects an iron-binding mechanism (*p* = 0.047248; MutPred2 [75]). There are 71 individuals heterozygous for this variant in gnomAD, but to the best of our knowledge, this is the first individual homozygous for the *ALMS1* duplication. Although the clinical characteristics described for this patient match the known features of Alström syndrome and only mutations in the *ALMS1* gene have been found to cause Alström syndrome, the homozygous VUS in *ALMS1* identified in the primary findings is technically considered a negative genetic result. However, we believe that it is important to present this case if the mutation becomes reclassified.

#### 3.2.2. *GNAT1*

**Figure 2:**
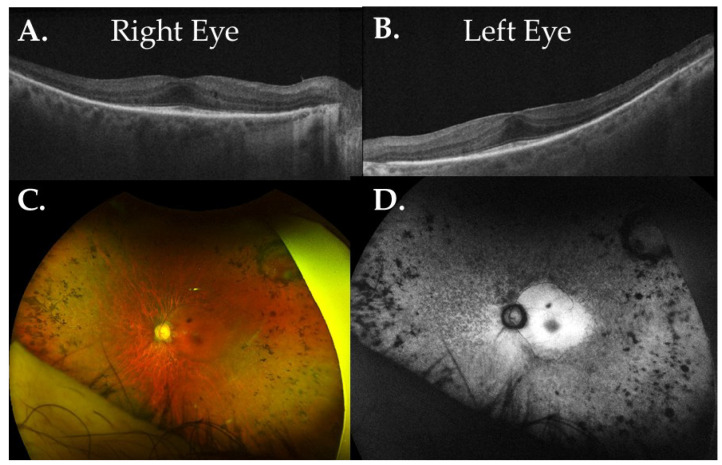
A patient with autosomal recessive RP had novel homozygous variant in *GNAT1*. (**A**,**B**) GP2-27 images revealed preservation of the fovea, including the outer nuclear layer and ellipsoid zone, with adjacent outer retinal degeneration. (**C**,**D**) Patient’s left eye has vascular attenuation, faint RPE changes (**C**), bone-spicule-like pigmentation, and peripapillary atrophy (**D**).

Patient GP2-27 experienced nyctalopia since birth. In his sixties, he began noticing a significant loss of peripheral vision. SD-OCT images support photoreceptor preservation of the fovea, including the outer nuclear layer and ellipsoid zone, with adjacent outer retinal degeneration (Figure 2A,B). Fundus imaging showed macular preservation (Figure 2C), peripapillary atrophy, and a perifoveal hyper-AF ring within the arcades (Figure 2D). Genetic testing for GP2-27 identified a novel homozygous variant c.758_771delinsC, p.Arg253Pfs*56 in *GNAT1.* This nonsense variant generates a frameshift resulting in a premature stop codon in the last exon and is absent in gnomAD (Table 2). This is not expected to result in nonsense-mediated decay, although it is predicted to truncate the protein and may result in disrupted protein function. Other downstream variants in *GNAT1*-related phenotypes have also been predicted to result in protein truncation. In summary, a novel homozygous pathogenic variant in *GNAT1* was found in a patient with early onset but late progressing autosomal recessive RP.

#### 3.2.3. *RAX2*

**Figure 3:**
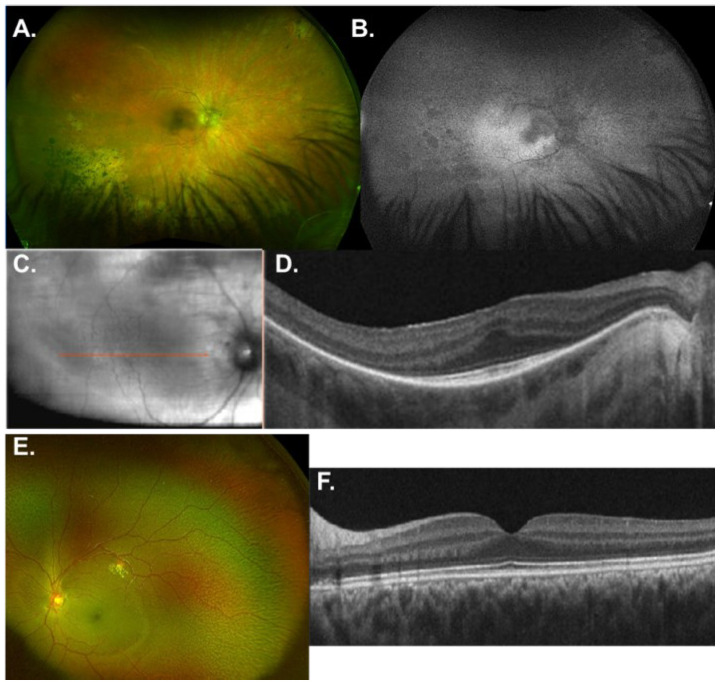
Imaging for patients with *RAX2* (**A**–**D**) or *RDH5* (**E**,**F**) variants. On Optos imaging, there was (**A**) chorioretinal atrophy and bone spicule pigmentation in the periphery for GP2-81 and (**B**) a hyper-autofluorescence signal within the macula. (**C**) The near-infrared image of scan location (red arrow) for GP2-81, indicating placement of the (**D**) horizontal line scan that showed foveal hypoplasia and EZ preservation in the foveal region. (**E**,**F**) GP2-82 *RDH5* had yellow punctae throughout the peripheral retina (**E**) and a normal macula on OCT (**F**).

GP2-81 is a 51-year-old male who was diagnosed with autosomal recessive RP at the age of 20 and hearing loss that began in the third decade of life. He had a fraternal twin brother who passed away in his 20s. No other family members were reported to have retinal disease. On Optos imaging, there was chorioretinal atrophy and bone spicule pigmentation in the periphery for GP2-81 (Figure 3A) as well as a hyper-autofluorescence signal within the macula (Figure 3B). The near-infrared image shows the scan location (Figure 3C) for the SD-OCT image that revealed foveal hypoplasia with the continuation of the inner retinal layers over the foveal region (Figure 3D). The EZ was preserved in the foveal region. Sequence analysis identified a heterozygous pathogenic variant *RAX2* c.236G>A, p.(Arg79Gln) [76] and a novel heterozygous VUS *RAX2* c.181G>A, p.(Ala61Thr). There are two individuals heterozygous for this VUS in gnomAD, and it is predicted to be deleterious by all in silico tools used (Table 2). This sequence change replaces alanine, which is neutral and non-polar, with threonine, which is neutral and polar, at codon 61 of the RAX2 protein (p.Ala61Thr). The pathogenicity score by MutPred2 [75] was 0.618, and the predicted ELM [77] and PROSITE motif alterations include the functional site for SH3 domain ligands (ELME000155), Protein kinase C phosphorylation site (PS00005), and HOMEOBOX_1 (PS00027). The affected molecular mechanisms are predicted to include a gain of the allosteric site at R57 (0.28 probability; *p* = 4.0 × 10^−3^; MutPred2 [75]) and altered metal binding (0.17 probability; *p* = 0.04). This variant has not been reported in the literature in individuals affected with *RAX2*-related conditions.

#### 3.2.4. *RDH5*

A 7-year-old boy, GP2-82, was diagnosed with fundus albipunctatus. There were white/yellow deep, fleck-like punctate lesions in the peripheral retina (Figure 3E), and the SD-OCT image (Figure 3F) was unremarkable. There is no known family history of a similar disease. Genetic testing detected a heterozygous pathogenic frameshift variant *RDH5* c.718dup, p.(Ala240Glyfs*19) [78,79,80,81] and a novel heterozygous missense variant *RDH5* c.833G>A, p.(Arg278Gln) [82]. Genetic testing of the patient’s biological parents found the variants to be in trans and were classified as likely pathogenic. *RDH5* c.833G>A, p.(Arg278Gln) is rare in control populations and predicted to be deleterious by in silico tools (Table 2). The MutPred2 modeling score was 0.659 and showed that the amino acid residue 278 is an ELM [77] di-arginine retention/retrieving signal motif (ELME000012) required for proper localization of the RDH5 enzyme within the RPE. Together with the Ala240Glyfs*19 mutation, there is likely a shortage of 11-cis retinal, resulting in the accumulation of 11-cis retinol, which contributes to the formation of the white-yellow flecks that are characteristic of fundus albipunctatus for patient GP2-82 (Figure 3E).

## 4. Discussion

We tested 103 individuals in this study and found causative mutations in 29 genes for 68% of patients tested. The genetic data facilitated the identification of 20 novel variants, six of which were reclassified from VUS to likely pathogenic by a certified geneticist BpG after familial segregation analysis (§; Table 1). The diagnostic yield of this study aligns with the range (56–76%) identified in previous studies, with mutation frequency depending on the region in which data was collected [18,19,40,83,84]. *ABCA4*-related disease is the most common cause of IRD [4,85,86]. Therefore, it is not surprising that this gene had the highest variant frequency in this cohort (28%, n = 12 patients, Figure 1A). This was similar to our previous study that found 23% (13 of 48 solved cases) [59]. Additionally, the frequency of *PRPH2*-related IRD remained the second-highest cause of IRD in this cohort, consistent with our previous findings [59].

Thirty-two percent of patients in this cohort received negative genetic testing results despite having a clinical IRD diagnosis. Three underlying possibilities may help to explain the inconsistencies between the clinical diagnosis and negative genetic testing. (1) The genetic landscape of IRDs is highly complex, with potential genes that have not been discovered. Additionally, next-generation sequencing (NGS) technologies, like the one used by BpG to generate the genetic reports for this study, might not detect all types of genetic variations, such as deep intronic mutations, large deletions or duplications, or epigenetic changes, which may also contribute to IRDs. (2) Another possibility is that some patients may have variants that are not yet documented in existing genetic databases and/or have not been assigned pathogenicity. For example, variants associated with late-onset conditions are likely to be more prevalent in the population due to their lower penetrance, making them harder to identify in bioinformatics analysis. In addition, the historic underrepresentation of non-white racial and ethnic groups in medical research and genomic databases contributes to a higher rate of negative genetic results when compared to White patients [87,88]. (3) There are certain acquired diseases that mimic IRDs. It is possible that patients with late-onset IRD and negative genetic results did not disclose complete medical history and could have instead acquired an IRD-mimicking disorder. These include toxic retinopathies, uveitis, autoimmune retinopathies, and unknown diseases with retinal involvement. Thus, improving sequencing technologies, methods for evaluating variant pathogenicity, increasing diversity in genomic databases, and using clinical and experimental data in tandem will likely reduce the unsolved cases in IRDs.

The remainder of this discussion will focus on the novel variants identified in the genes *ALMS1*, *GNAT1*, *RAX2*, and *RDH5.* These variants are associated with ultra-rare conditions that have either not been documented in the literature or only a few cases have been identified.

Alström syndrome is an autosomal recessive genetic disorder that has a prevalence of about 1 per 1,000,000 individuals and is considered ultrarare [89]. It is characterized by the progressive development of multi-organ pathologies such as cone-rod dystrophy, hearing loss, childhood truncal obesity, insulin resistance and hyperinsulinemia, type 2 diabetes, hypertriglyceridemia, short stature in adulthood, cardiomyopathy, and progressive pulmonary, hepatic, and renal dysfunction. As of this manuscript, patient GP2-41 did not report renal, pulmonary, or cardiac involvement. However, this is a progressive disease with the evolution of clinical features that vary by individual, often making diagnosis difficult. Although the genetic results were negative for GP2-41, we highlight this case as the findings may aid in the early diagnosis of future patients with Alström syndrome. Mutations in the first four exons of *ALMS1* are thought to be embryonic lethal [90]. However, the homozygous six-base pair duplication identified in our patient occurs in exon 2 and may represent a milder form of Alström syndrome. Also, it could be that this *ALMS1* variant is not pathogenic, and the symptoms are part of an undetermined diagnosis. Nevertheless, the parents received counseling and guidance from a medical geneticist coordinating with the medical specialties involved in their son’s care.

The identification of the homozygous *GNAT1* variant c.758_771delinsC, p.(Arg253Profs*56) in a patient with early-onset but late-progressing autosomal recessive RP (arRP) provides further support for the role of *GNAT1* in retinal dystrophies. *GNAT1* gene mutations are traditionally linked to congenital stationary night blindness (CSNB) [91,92,93], but two cases of arRP have been reported [94,95]. *GNAT1* encodes the alpha subunit of transducin, a crucial protein involved in the phototransduction pathway. More specifically, it is involved in the coupling of rhodopsin with cGMP-phosphodiesterase during rod photoreceptor signaling. The variant results in a frameshift, creating a premature stop codon and leading to truncation of the protein. This truncation likely disrupts key functional domains, including the GTP and PDEγ binding sites and the interaction motif with rhodopsin at the C-terminus, all of which are essential for proper phototransduction. Similar to other truncating *GNAT1* variants, such as p.(Gln302*) [94] and p.(Cys321*) [95], which have been associated with lifelong night blindness and late-onset RP, the p.(Arg253Profs*56) variant likely results in a loss of function of the transducin alpha subunit. Previous reports of *GNAT1* knockout mice models [96] and human cases have shown a slow degeneration of rod photoreceptors, consistent with the delayed progression observed in this patient. In these cases, the phenotype is characterized by early night blindness, which can manifest in childhood or early adulthood, with later onset of more severe retinal degeneration. A golden appearance of the fundus without the Mizuo-Nakamura phenomenon was found in the patients in the study conducted by Kubota et al. [97]. In the context of patient GP2-34, the early onset of night blindness followed by slow progression of RP is consistent with other *GNAT1* truncating mutations. The mechanism of action is likely a loss of functional transducin, impairing the phototransduction process and leading to progressive rod degeneration over time. Given the similar phenotypes observed in patients with truncating *GNAT1* variants, including p.(Gln302*) [94] and p.(Cys321*) [95], the p.(Arg253Profs*56) variant likely follows a similar pathogenic pathway, confirming its role in the development of early-onset arRP with late progression.

*RAX2*, a transcription factor essential for photoreceptor gene regulation during late retinogenesis, has been associated with nonsyndromic arRP (RP86) recently (2019) [98]. Only five other patients with mutations in *RAX2* have been described to date [98]. The age of onset varied from childhood to mid-40s, with an average around mid-30s. Previously reported biallelic *RAX2* variants have been linked to loss of function mutations due to disruption of key protein domains, leading to phenotypes that align with our patient’s (GP2-91) clinical presentation. The novel variant p.(Ala61Thr) is located in the highly conserved homeodomain of RAX2, which is critical for DNA binding and proper transcriptional regulation. Protein structure modeling of other recessive RAX2 variants, such as p.(Ser49Pro) and p.(Pro52Arg), has demonstrated that mutations in this region can severely disrupt protein folding and stability, resulting in loss of function [98]. Given that both p.(Arg79Gln) and p.(Ala61Thr) occur within this same domain, it is plausible that these variants impair *RAX2* function in a similar manner, leading to the patient’s retinal degeneration. The p.(Arg79Gln) variant in particular, located close to the previously modeled dominant p.(Arg87Gln) variant [98], may interfere with DNA-binding properties and disrupt higher-order complex formation of *RAX2* with other transcription factors like CRX, although its exact impact on protein stability is uncertain. In contrast, the effects of the p.(Ala61Thr) variant are less well characterized, though it is reasonable to assume that this change could similarly alter *RAX2*’s ability to interact with DNA or its binding partners. Foveal hypoplasia (Figure 3D) has not been reported in the literature associated with *RAX2*, but it can be seen in the SD-OCT images of patients from Van de Somplele et al. [98].

*RDH5* encodes 11-*cis* retinol dehydrogenase, a NAD-dependent dehydrogenase enzyme belonging to the short-chain dehydrogenases/reductases (SDR) family. Its expression is abundant in RPE, where it resides in the smooth endoplasmic reticulum [99] and catalyzes the final step in the biosynthesis of 11-*cis* retinaldehyde [100,101,102], the universal chromophore of visual pigments. This enzyme is also able to oxidize 9- and 13-*cis*-retinols, but not all-trans-retinols. Defects in the *RDH5* gene cause autosomal recessive FA, a rare form of flecked retina disease that is characterized by impaired night vision and uniformly distributed small yellow-white dots in the retina. The disease is stationary in most cases, but progressive cone dystrophy and macular atrophy can occur. A total of 56 different *RDH5* mutations are currently reported in the literature, 20 of which have been reported to be associated with macular changes. Pathogenic variants in the *RDH5* gene are the most common cause of fundus albipunctatus, which may be accompanied by macular dystrophy, bull’s eye maculopathy, photophobia, and RPE degeneration [103]. Patients with macular atrophy, which is more commonly recognized in subjects with biallelic *RDH5* mutations, have lifelong night vision difficulties, with the likelihood of deterioration in central vision increasing with age [99,104,105,106]. The di-Arg motif is present on the cytosolic side of many transmembrane proteins and serves as an ER-retrieval and retention motif. Disruption of this motif would lead to protein mislocalization away from the ER, the site where it oxidizes 11-*cis*-retinol to 11-*cis*-retinal. These mutations in the *RDH5* gene may reduce or eliminate the function of the enzyme, leading to a shortage of 11-*cis*-retinal and impaired vision. In addition, the buildup of 11-*cis*-retinol would be toxic to the RPE and can be seen as the white-yellow flecks on fundus imaging (Figure 3E).

The presence of VUS and the need for family-based studies to phase variants and confirm pathogenicity are commonly reported challenges in genetic research involving patients with IRD. Recommendations by the American College of Medical Genetics and Genomics (ACMG) [107] indicate that VUS should not be used for clinical decision-making. Instead, medical decisions should be based on a patient’s personal and family history. Because co-segregation analyses are needed to gather more evidence for variant re-classification, this work is ongoing as we continue to collect additional genetic testing from family members to clarify inheritance patterns and establish genotype–phenotype correlations.

Our research expands the understanding of the genetic basis of IRD and underscores the clinical and genetic heterogeneity of these conditions, highlighting the need for personalized genetic screening and targeted therapeutic strategies.

## Figures and Tables

**Table 1:** Genetic testing results for cohort.

ID	Age at Onset/Visit	Race (Self-Reported)	Sex	Inheritance	IRD DX (Syndrome)	Gene	Variant(s)
GP2-1	6/23	W	F	R	STGD1	*ABCA4*	c.5461-10T>C/c.179C>T p.(A60V)/c.5603A>T p.(N1868I)
GP2-2	15/53	W	F	R	STGD1	*ABCA4*	c.5461-10T>C/c.5056G>A p.(V1686M)/c.5603A>T p.(N1868I)
GP2-3	26/33	W	M	R	STGD1	*ABCA4*	c.4253+43G>A; c.2041C>T p.(R681*)
GP2-4	53/63	W	F	R	STGD1	*ABCA4*	c.5461-10T>C/c.3113C>T p.(A1038V)/c.5603A>T p.(N1868I)
GP2-5	54/61	W	M	R	STGD1	*ABCA4*	c.4685T>C p.(I1562T)/c.4234C>T p.(Q1412*)
GP2-6	10/35	W	F	R	CD/CRD	*ABCA4*	c.5882G>A p.(G1961E)/c.3113C>T p.(A1038V)/c.1622T>C p.(L541P)
GP2-7	10/34	W	F	R	CD/CRD	*ABCA4*	c.4539+2028C>T/c.634C>T p.(R212C)
GP2-8	29/69	W	F	R	MD	*ABCA4*	c.5603A>T p.(N1868I)/c.5734G>A p.(E1912K) ‡
GP2-9	25/35	NA	M	R	STGD1	*ABCA4*	c.6079C>T p.(L2027F)/c.1292G>A p.(W431*)
GP2-10	32/35	W	F	R	STGD1	*ABCA4*	c.6317G>A p.(R2106H)/c.5714+5G>A
GP2-11	32/32	W	M	R	STGD1	*ABCA4*	c.6079C>T p.(L2027F)/c.768G>T p.(V256=)
GP2-12	23/31	W	F	R	STGD1	*ABCA4*	c.6166A>T p.(K2056*)/c.2588G>C p.(G863A)/c.5603A>T p.(N1868I)
GP2-41	11/11	W	M	R	RP (Alström)	negative	***ALMS1* c.103_108dup p.(A35_A36dup)/c.103_108dup p.(A35_A36dup)** ‡
GP2-13	9/47	W	M	R	RP (BBS)	*BBS1*	c.1169T>G p.(M390R)/c.1169T>G p.(M390R)
GP2-14	10/54	W	F	R	RP (BBS)	*BBS1*	c.1169T>G p.(M390R)/c.1169T>G p.(M390R)
GP2-15	11/37	W	F	R	RP (BBS)	*BBS1*	c.1169T>G p.(M390R)/c.1169T>G p.(M390R)
GP2-16	12/41	W	F	R	RP (BBS)	*BBS1*	c.1169T>G p.(M390R)/**c.800G>T p.(C267F)** ‡
GP2-103	8/18	W; A	F	R	RP (BBS)	*BBS10*	c.1677C>G p.(Y559*)/**c.1121T>C p.(L374P)** ‡
GP2-17	3/10	NA	M	R	RP (BBS)	*BBS4*	c.332+8T>C/**c.1248G>C p.(E416D)** §
GP2-18	4/12	W	M	D	Best	*BEST1*	c.652C>T p.(R218C)
GP2-19	7/7	W	M	D	Best	*BEST1*	**c.878A>G p.(Q293R)**
GP2-20	10/32	W	M	D	Best	*BEST1*	c.275G>A p.(R92H)
GP2-21	27/28	W	M	D	Best	*BEST1*	c.652C>A p.(R218S)
GP2-22	8/45	W	F	R	Best	*BEST1*	c.422G>A p.(R141H)/c.602T>C p.(I201T)
GP2-23	0/81	H	F	R	RP	*CNGB1*	c.2957A>T p.(N986I)/**c.2396T>G p.(L799R)** §
GP2-24	3/19	W	F	R	RP	*CRB1*	c.2843G>A p.(C948Y)/c.3014A>T p.(D1005V) §
GP2-39	63	W	M	S	MD	negative	***CTNNA1* chr5:g.138117546-138473144dup** ‡
GP2-44	30/64	W	F	R	RP	negative	***DRAM2* c.328G>A p.(A110T)/c.328G>A p.(A110T)** ‡
GP2-35	6/6	W	M	S	RP	*DYNC2H1*	c.7156G>T p.(G2386*)/**c.10205_10207del p.(T3402del)** ‡
GP2-25	28/74	W	M	R	RP	*EYS*	c.7095T>G p.(Y2365*)/c.1505_1506insGA p.(F502Lfs*14) §
GP2-104	20/24	W	M	R	RP	*EYS*	c.2528G>A p.(G843E)/c.(3443+1_3444-1)_(6424+1_6425-1)del encompassing exons 23–31
GP2-26	20/21	H	F	D	FEVR	*FZD4*	c.313A>G p.(M105V)
GP2-27	0/68	H	M	R	RP	*GNAT1*	c.758_771delinsC p.(R253Pfs*56)
GP2-34	1/41	B	M	D	CD/CRD	*GUCY2D*	**c.2578T>C p.(S860P)** ‡
GP2-107	5/28	W	M	D	RP	*IMPDH1*	c.931G>A p.(D311N)
GP2-28	10/41	W	F	S	FEVR	*KIF11*	c.308+1G>A
GP2-29	16/33	W	F	MT	RP	negative	*MT-ND4* m.11778G>A (100%)
GP2-30	42/45	W	F	MT	PD (MMSD)	*MT-TL1*	m.3243A>G (15.3%)
GP2-31	60/61	W	F	MT	MD (MSMD)	*MT-TL1*	m.3243A>G (28.8%)
GP2-32	21/49	W	F	MT	RP (MSMD)	*MT-TL1*	m.3255G>A (24.8%)
GP2-33	0/57	W	F	R	RP (USH1)	*MYO7A*	c.1559_1560del p.(T520Rfs*26)/c.5302C>T p.(Q1768*)
GP2-37	13/13	NA	F	S	MD	negative	
GP2-40	10/61	NA	F	D or XL	MD	negative	
GP2-43	25/36	NA	F	S	RP	negative	
GP2-45	32/40	W	M	S	RP	negative	OPA1 c.2708_2711del p.(V903Gfs*3)
GP2-46	50/60	W	F	S	RP (Stickler)	negative	
GP2-47	60/60	H	F	S	RP	negative	
GP2-48	62/68	A	F	S	RP	negative	
GP2-49	12/32	H	F	S	RP (USH2)	negative	
GP2-50	31/43	B	M	S	RP	negative	
GP2-51	50/58	W	F	S	RP	negative	
GP2-52	9/20	W	M	S	MD	negative	
GP2-53	61/61	NA	M	S	MD	negative	
GP2-54	37/43	W	F	S	MD	negative	
GP2-55	28/39	NA	M	D or XL	RP	negative	
GP2-56	16/54	W	F	S	RP	negative	
GP2-57	25/54	A	F	S	RP	negative	
GP2-58	30/67	NA	M	S	RP	negative	
GP2-59	35/75	W	F	S	RP	negative	
GP2-60	40/65	W	M	S	RP	negative	
GP2-61	45/56	H	M	D or XL	RP	negative	
GP2-62	48/55	NA	F	S	RP	negative	
GP2-63	51/55	W	F	S	RP	negative	
GP2-64	5/24	W	M	S	RP	negative	
GP2-65	46/66	W	M	S	STGD1	negative	
GP2-66	59/78	W	F	R	STGD1	negative	ABCA4 c.5603A>T p.(N1868I)
GP2-67	59/63	W	M	S	XLRS	negative	
GP2-100	30/60	H	M	S	RP	negative	
GP2-101	34/40	W	F	S	MD	negative	
GP2-106	0/36	B	M	S	MD	negative	
GP2-68	6/8	NA	F	R	MD	*NMNAT1*	c.507G>A p.(W169*)/c.709C>T p.(R237C)
GP2-69	8/47	W	F	R	RP	*NR2E3*	c.119-2A>C; c.767C>A p.(A256E)
GP2-70	13/21	B	M	D	MD	*PROM1*	c.1117C>T p.(R373C)
GP2-71	6/39	W	M	D	RP	*PRPF31*	c.(238+1_239-1)_(1374+1_1375-1)dup exons 4–13
GP2-72	25/60	W	M	D	RP	*PRPF31*	c.697+1G>C
GP2-73	38/48	W	F	D	RP	*PRPF31*	c.1031_1032del p.(P344Rfs*130)
GP2-74	9/71	W	M	D	MD	*PRPH2*	c.907_910delinsCG p.(S303Rfs*88)
GP2-75	40/60	NA	M	D	MD	*PRPH2*	c.629C>G p.(P210R)
GP2-76	40/43	B	M	D	MD	*PRPH2*	c.515G>A p.(R172Q)
GP2-77	45/63	W	M	D	MD	*PRPH2*	c.828+3A>T p.(?)
GP2-78	53/71	W	M	D	PD	*PRPH2*	c.(828+1_829-1)_(1*_?)del
GP2-79	24/56	W	M	D	RP	*PRPH2*	c.828+3A>T, p(?)
GP2-80	53/82	W	F	D	MD	*PRPH2*	**c.620A>G p.(D207G)**
GP2-81	20/51	W	M	R	RP	*RAX2*	c.236G>A p.(R79Q)/c.181G>A p.(A61T) ‡
GP2-82	7/7	H	M	R	FA	*RDH5*	c.718dup p.(A240Gfs*19)/**c.833G>A p.(R278Q)** §
GP2-83	11/33	NA	F	D	RP	*RHO*	c.509C>G p.(P170R)
GP2-84	13/34	W	F	D	RP	*RHO*	c.152G>T p.(G51V)
GP2-85	10/45	A	M	D	RP	*RHO*	**c.404_405delinsTT p.(R135L)**
GP2-86	39/42	NA	F	S	RP	*RP2*	**c.969G>C p.(K323N)** ‡
GP2-87	7/7	A	F	R	CD/CRD	*RPE65*	c.1102T>C p.(Y368H)/c.718G>T p.(V240F)
GP2-88	10/30	H	F	R	RP	*RPE65*	c.1067dup p.(N356Kfs*9)/c.1067dup p.(N356Kfs*9)
GP2-89	0/61	W	M	XL	MD	*RPGR*	c.3178_3179del p.(E1060Rfs*18)
GP2-90	3/5	H	F	XL	RP	*RPGR*	c.2362G>T p.(E788*)
GP2-91	12/23	NA	M	XL	RP	*RPGR*	c.2146G>T p.(E716*)
GP2-92	5/35	H	M	XL	XLRS	*RS1*	c.208G>A p.(G70S)
GP2-93	20/20	W	M	XL	XLRS	*RS1*	CNV c.(52+1_53-1)_(78+1_79-1)del
GP2-94	16/73	W	M	R	RP	*USH2A*	c.12295-2A>G/c.10073G>A p.(C3358Y)
GP2-95	26/28	H	M	R	RP (USH2)	*USH2A*	c.1000C>T p.(R334W)/c.1000C>T p.(R334W)
GP2-96	54/55	B	M	R	RP	*USH2A*	c.12145G>A p.(A4049T)/**c.274T>G p.(S92A)** ‡
GP2-97	50/72	NA	M	R	RP	*USH2A*	**c.2384G>A p.(C795Y)**/c.11712-3520_11712-3519ins[GTAAGGATTCCCAATAATCCTTACCCT]
GP2-98	30/46	W	M	R	RP (USH2)	*USH2A*	c.(5298+1_5299-1) (5572+1_5573-1)del/c.14851T>G p.(W4951G) ‡, **c.253A>T p.(I85F)** §
GP2-99	2/40	W	F	R	RP (USH2)	*USH2A*	c.13374del p.(E4458Dfs*3)/c.12152_12153insTT p.(E4051Dfs*2)
GP2-105	17/33	B	M	R	RP (USH2)	*USH2A*	c.6428del p.(P2143Qfs*9)/**c.4001A>G p.(K1334R)** ‡

White (W), Black (B), Native American (NA), Hispanic (H), Asian (A); male (M), female (F); dominant (D), recessive (R), isolate (S), mitochondrial (MT), X-linked (XL), unknown (UNK); retinitis pigmentosa (RP), cone dystrophy/cone-rod dystrophy (CD/CRD), Stargardt Disease (STGD1), macular dystrophy (MD), fundus albipunctatus (FA), X-linked retinoschisis (XLRS), Leber congenital amaurosis (LCA), choroideremia (CHM), Best disease (Best), familial exudative vitreoretinopathy (FEVR), Bardet–Biedl Syndrome (BBS), Usher syndrome type II (USH2), Usher syndrome type I (USH1), pattern dystrophy (PD), multiple mitochondrial dysfunctions syndrome (MMDS); Mutation: novel (Bold), VUS (‡), Familial segregation/alleles in trans (§).

**Table 2:** Variant modeling and pathogenicity data for novel variants identified.

Gene (Inheritance)	Variant	gnomAD Allele Frequency	PolyPhen	SIFT	MUT-TASTER	Mut-Pred2	MutPred2 Mechanisms *p* < 0.05	ACMG Criteria
*ALMS1 (Rhom)*	c.103_108dup p.(A35_A36dup)	0.0005103	N/A	N/A	N/A	0.29022	Iron binding	PP4, BP3
*BBS1 (Rhet)*	c.800G>T p.(C267F)	0	PrD	D	DC	0.925	Altered DNA binding, altered transmembrane protein, loss of disulfide linkage at C267	PM2, PP3, PP4
*BBS4 (Rhet)*	c.1248G>C p.(E416D) §	0	PoD	T	DC	0.411	None	PM2, PP3, PP4, PM3
*BBS10 (Rhet)*	c.1677C>G p.(Y559*)	0	PrD	D	DC	0.52879	N/A	PM2, PVS1, PP4
*BBS10 (Rhet)*	c.1121T>C p.(L374P)	0	PrD	D	DC	0.484	None	PM2, PP4
*CTNNA1 (d)*	chr5:g.138117546-138473144dup	0	N/A	N/A	N/A	0.37257	N/A	PM2
*DRAM2 (Rhom)*	c.328G>A p.(A110T)	3.188 × 10^−5^	PrD	D	DC	0.744	Loss of helix, gain of strand, altered transmembrane protein	PP3
*DYNC2H1 (Rhet)*	c.10205_10207del p.(T3402del)	0.000125	N/A	N/A	N/A	0.45459	Iron-binding; catalytic site	PM2
*GNAT1 (Rhom)*	c.758_771delinsC p.(Arg253Pfs*56)	0	N/A	N/A	N/A	0.40539	N/A	PM2, PM4
*GUCY2D (d)*	c.2578T>C p.(S860P)	3.978 × 10^−6^	PrD	D	DC	0.597	Altered coiled-coil, altered transmembrane protein	PP3
*PRPH2 (d)*	c.907_910delinsCG p.(S303Rfs*88)	0	N/A	N/A	N/A	0.31587	N/A	PM2, PM4
*PRPH2 (d)*	c.620A>G p.(D207G)	0	PrD	D	DC	0.836	Altered transmembrane protein, loss of proteolytic cleavage at R203	PM2, PP3
*RAX2 (Rhet)*	c.181G>A p.(A61T)	8.326 × 10^−6^	PrD	D	DC	0.618	Gain of allosteric site at R57, metal-binding	PP3
*RDH5 (Rhet)*	c.833G>A p.(R278Q) §	2.386 × 10^−5^	PrD	D	DC	0.659	Altered metal-binding, gain of catalytic site at Y281	PM3, PP3
*RHO (d)*	c.404_405delinsTT, p.(Arg135Leu)	0	PrD	D	DC	0.941	Loss of strand, gain of helix, loss of disulfide linkage at C140, altered ordered interface, altered transmembrane protein	PM2, PP3
*RP2 (x)*	c.969G>C p.(K323N)	0	B	T	DC	0.43	None	PM2, PP3, BP4
*USH2A (Rhet)*	c.274T>G p.(S92A)	7.971 × 10^−6^	B	D	DC	0.216	None	PP4, PP3, PP4
*USH2A (Rhet)*	c.253A>T p.(I85F) §	0	PrD	D	DC	0.328	None	PM2, PP3, PM3
*USH2A (Rhet)*	c.4001A>G p.(K1334R)	7.081 × 10^−6^	B	T	PM	0.105	None	PP4, BP4
*USH2A (Rhet)*	c.2384G>A p.(C795Y)	0	PrD	D	DC	0.921	Altered transmembrane protein, gain of disulfide linkage at C792, altered metal binding, gain of catalytic site at C795, gain of sulfation at C795	PM2, PP3, PP4

Genome Aggregation Database (gnomAD), Polymorphism Phenotyping (PolyPhen), Sorting Intolerant from Tolerant algorithm (SIFT), MutationTaster (MUTTASTER), American College of Medical Genetics (ACMG), absent from controls (or at extremely low frequencies if recessive) in Exome Sequencing Project, 1000 Genomes Project, gnomAD, or Exome Aggregation Consortium (PM2), multiple lines of computational evidence support a deleterious effect on the gene or gene product (PP3), patient’s phenotype or family history is highly specific for a disease with single genetic etiology (PP4), in-frame deletions/insertions in a repetitive region without a known function (BP3), multiple lines of computational evidence suggest no impact on gene or gene product (BP4), for recessive disorders, detected in trans with a pathogenic variant (PM3), protein length changes as a result of in-frame deletions/insertions in a nonrepeat region or stop-loss variants (PM4), null variant (nonsense, frameshift, catonical ± 1 or 2 splice sites, initiation codon, single or multiexon deletion) in a gene were loss of function is a known mechanism of disease (PVS1), possibly damaging (PoD), probably damaging (PrD), benign (B), recessive heterozygous but paired with a second pathogenic or likely pathogenic mutation in the same gene (*Rhet*), recessive homozygous (*Rhom*), dominant (*d*), x-linked (*x*), tolerated (T), deleterious (D), disease causing (DC), polymorphism (PM), not applicable (N/A).

## Data Availability

NGS panel statistics are available upon request.

## Supplementary Materials

The following supporting information can be downloaded at: https://www.mdpi.com/article/10.3390/genes16010032/s1, Table S1: Clinical Characteristics associated with novel variants.
