# Supplementary material for: Expanding the Mutation Spectrum for Inherited Retinal Diseases
Source: Genes (Basel). 2024 Dec 28;16(1):32. doi: 10.3390/genes16010032 (PMC11764958; doi:10.3390/genes16010032)
Supplement: Supplementary file 1 [file genes-16-00032-s001.zip › genes-3350988-supplementary.pdf]

Supplemental Table S1. Clinical Characteristics associated with novel variants

GP2-103	Patient is an 18 year old female diagnoses with RP associated with BBS (Laurence-Moon) at age 8. Left extra digit removal at age 6 months. History of liver cirrhosis, ovarian hyperthecosis with hyperandrogenemia, obesesity, hypothyroidism, hypertension, proteinuria, diabetes, cataract, asthma, GERD, and scoliosis, thoracic region. No family history of similar disease. Nyctalopia, delayed dark adaptation, and progressive loss of peripheral vision beginning at age 3 years. Loss of central vision beginning at age 5 years. Attenuated vessels, pigment mottling, and pallor of the optic disc.
GP2-16	Patient is a 41-year-old female with RP, diagnosed in 1993, associated with BBS (Laurence-Moon). Patient had extra digits removed at age 2. Clinical features include posterior subcapsular polar age-related cataract, OU, arterial attenuation, waxy pallor of the optic disc, and RPE mottling/bone spicules. Associated medical conditions include: history of migraine headache(s); rare ovarian cancer; immature teratoma/epidymoma and a history of Bell's palsy, malignant neoplasm metastatic to peritoneum. BCVA: HM OD and OS. There is no reported family history of similar disease.
GP2-17	Patient is a 10 year old boy diagnosed with RP. Night blindness began at age 3 years and delayed dark adaption beginning at age 4 years. Obese, ASD. Foveal hypopigmentation with stippled RPE changes. Both eyes showed faint EZ irregularities in the parafovea OU. Optos widefield showed blunted foveal light reflex with hypopigmentation OU with stippled parafoveal hyperAF.
GP2-19	Patient is a 7-year-old boy with Best disease diagnosed in 2022, BCVA measured in 20 feet 20/63 OU. Large organized yellow vitelliform lesion, OU and bilateral hypermetropia. No significant medical non-ocular issue. No known family history.
GP2-23	Patient is an 81-year-old female with RP diagnosed in 1991. Presence of intraocular lens. History of night blindness, reduced peripheral (side) vision loss, has had cataract, problem with dark adaptation. Disk pallor, PVD, arteriolar narrowing, flat macula, bone spicule pigmentation. BCVA measured in 20 feet 20/32 OD and 20/40 OS. History of diabetes. Mother diagnosed RP.
GP2-25	Patient is a 74-year-old male with RP, diagnosed at age 28. Pseudophakia, arterial attenuation, RPE changes, bone-spicule pigmentation, waxy pallor of optic disc, and corneal edema. Kidney anomaly. BCVA: HM OD, 20/125 OS. No known family history.
GP2-27	Patient is a 68 year old man diagnosed with RP. Central foveal EZ island. Nyctalopia and delayed dark adaptation since childhood. Hyperlipidemia, hypertension, pseudophakia, chronic GERD, retinal edema of left eye, epiretinal membrane both eyes, posterior capsular opacification bilateral; Vascular attenuation, RPE changes, bone-spicule pigmentation, waxy pallor of optic disc
GP2-34	Patient is 41-year old male with cone-rod dystrophy. Decreasing acuity over past ~10y, color blindness, photophobia, decreased vision OU progressive since infancy. On no medications, no systemic findings. Dark adaptation problems since age 25. Diffuse thinning of retina, OU. Optos widefield showed blunted foveal reflex, no RPE changes. Disc pallor OU. Mother has Mother with similar condition, similarhyperAF, and same gene mutation
GP2-39	Patient is a 69-year-old male with macular dystrophy. Optos widefield showed round patches of RPE atrophy and depigmentation (no nasal involvement). Glaucoma, coronary artery disease, bypass surgery, hyperlipidemia, hypertension, pseudophakia. Adopted, no family history.
GP2-41	Patient is an 11-year-old male with RP, obesity, chronic superficial gastritis without bleeding, eosinophilic gastritis, fatty liver, GERD; developmental delay, microcephaly syndrome, OCD, sensory disorder. Adopted, no family history.
GP2-44	Patient is a 64-year-old female with macular disease diagnosed at 30 years of age and RP diagnosed at 50 years of age. Vitreous syneresis, waxy pallor of optic disc, RPE changes in the macula, arterial attenuation, RPE mottling/bone spicules. Associated medical conditions: Diabetes, Kidney cancer, history of left renal cyst, surgery of 20% left nephrectomy. BCVA: CF OD and HM OS. No known family history.
GP2-74	Patient is a 71 year old woman diagnosed with macular dystrophy at age 9 years. PVD; PCIOL; vascular attenuation; Diffuse atrophic changes in macula
GP2-80	Patient is a 82-year-old female with macular dystrophy diagnosed in 1992. She also has hearing deficit. BCVA: 20/63 OU. Father and sister with macular degeneration, reduced uncorrectable central vision at age 50 or younger.
GP2-81	Patient is a 51-year-old male with RP and hearing loss diagnosed at age 43 years old. Decreased peripheral vision, vitreous syneresis, waxy pallor of optic disc, RPE changes in the macula, macular edema, arterial attenuation RPE mottling/bone spicules. BCVA: OD: 20/63; OS: 20/50. History of high blood pressure.

GP2-82	Patient is a 7-year-old boy with fundus albipunctatus. BCVA: OD 20/32, OS 20/25. Macula flat OU, rare flecks at the arcades borders OU, white/yellow deep fleck-like punctate lesions in the periphery OU. There is no known family history of similar disease
GP2-85	Patient is a 45-year-old male with a diagnosis of RP since age 24. BCVA 20/1000 OU. Asteroid hyalosis, Disk pallor, arteriolar narrowing, RPH changes in the macula, flat macula, bone spicule pigmentation. Nyctalopia beginning at age 10 followed by visual field constriction at 16 years. Non-ocular medical history was unavailable. There is a family history of several paternal relatives with RP (male-to-male transmission) presenting significant peripheral vision loss, reduced, uncorrectable central vision at age 50 or younger, blindness at age 60 or younger and night blindness.
GP2-86	Patient is 42-year old female diagnosed with RP at age 39. PVD OU, Nuclear sclerosis OU, myopia of both eyes; Waxy pallor of disc, vascular attenuation, bone spicules, OU, delayed dark adaptation, nyctalopia, and reduced peripheral vision; surgery history of LASIK (laser assisted in situ keratomileusis); .
GP2-96	Patient is a 55-year-old male diagnosed with RP in 2021. Moderate Myopia. BCVA: 20/20 OU. Patient presents with mid-peripheral bone spicules, optic nerve pallor, vessel attenuation, hyper autofluorescence ring in the macula with preservation of macular photoreceptors, and visual field constriction. Associated medical conditions: High Blood Pressure, Diabetes. Family history: aunt with blindness (unknown etiology).
GP2-97	Patient is a 72-year-old male with RP (2007). Waxy pallor of disc, vascular attenuation, bone spicules, vitreous syneresis, posterior subcapsular polar age-related cataract, nyctalopia, and contraction of visual field. BCVA measured in 20 feet 20/63 OD and 20/200 OS. The patient also has high blood pressure and Crohn's disease. There is no reported family history of a similar disease.
GP2-98	Patient is a 46-year-old male with RP and hearing deficit, diagnosed with Usher syndrome, type 2. Mild myopia and color blind. Waxy pallor of disc, vascular attenuation, bone spicules, and nyctalopia. BCVA (measured in 20 feet) is 20/50 OD and 20/40 OS. Other clinical features include visual field reduced to central 10 degrees and recent dizzy spells. Family history includes a sister with RP and hearing loss.

OCT, optical coherence tomography; AF, autofluorescence; GERD, Gastroesophageal reflux disease; OCD, obsessive compulsive disorder; BCVA, Best Corrected Visual Acuity; OU, oculus uterque; PCIOL, posterior chamber intraocular lens; PVD, posterior vitreous detachment; BBS, Bardet-Biedl Syndrome; HM, hand motion; OD, oculus dexter; OS, oculus sinister; ASD, autism spectrum disorder ; RPE, retinal pigment epithelium; EZ, ellipsoid zone; CF, counting fingers; ERM, epiretinal membrane;
